# The futility of searching for a single-best insemination method

**DOI:** 10.1007/s10815-020-01991-4

**Published:** 2020-11-03

**Authors:** Derek Keating, Gianpiero D. Palermo

**Affiliations:** The Ronald O. Perelman and Claudia Cohen Center for Reproductive Medicine, Weill Cornell Medicine, New York, NY USA

We recognize the effort of Geng et al. [1] for embarking on this endeavor. It is a well conducted analysis of the literature. However, the review fails to materially extend the vast amount of literature exploring the role of ICSI for the treatment of nonmale factor infertility.

While the authors identified 1597 records and screen 962, eventually only 26 were deemed worthy of further analysis. These reports were mostly represented by retrospective cohort studies with only 4 randomized controlled trials. While the outcome could have been biased by maternal age, the authors concluded that the use of ICSI insemination is not justified for nonmale factor infertility.

The analyses of the literature to try to identify an evidence-based justification for one procedure over the other in couples with normal gametes, in terms of fertilization and pregnancy outcome results, has often resulted in inconclusive outcome.

While comparison between standard in vitro insemination and ICSI procedures for patients with apparently normal gametes, and maternal age is controlled for, seems attractive to a reproductive reader, the comparison is not equitable. ICSI was not developed to be superior to standard in vitro insemination; it was designed to overcome shortcomings of IVF in certain patients. It entails higher operator skills because it is a more invasive procedure and therefore involves potential harm to the oocyte, including an arbitrary selection of ideal sperm to be injected. The procedure is also carried out on a microscope most often not within a laminar flow hood, therefore potentially exposing the gametes to temperature and pH fluctuations. ICSI, however, is extremely popular and remains so because it may represent a form of evolution of the in vitro insemination process.

The execution of this type of study is justified by an increase, up to 72% in 2012, in ICSI utilization with a major component related to nonmale factor infertility. This increment is observable in data from the Center for Disease Control, wherefore in 2016 couples diagnosed with male factor infertility experienced an ICSI utilization up to 94%, and ICSI utilization was also up to 72% for cases where male factor infertility was not a contributing factor [2]. The increase in utilization of ICSI has been shown to not correlate with an increase in a male factor infertility diagnosis and is not justified by a sizeable increase in live birth rate [3]. The proposed indication of the utilization of ICSI for nonmale factor infertility are poor quality oocytes, low oocyte yield, advanced maternal age, fertilization failure with standard in vitro insemination, PGT, IVM, or the insemination of previously cryopreserved oocytes. The evidence of studies of ICSI for nonmale factor indications do not evidence a superiority of ICSI are because indeed the two techniques aim for the same outcome—to achieve fertilization. If both the male and female gamete are healthy, these techniques perform the same. In this circumstance, ICSI cannot yield superior clinical outcome to standard in vitro insemination if both are carried out correctly in an ideal scenario. If one gamete is lacking, such as when a subtle male factor like DNA fragmentation is present, ICSI may help obviate the problem by providing more consistent fertilization, embryo development, and implantation.

People have maybe forgotten why ICSI was developed. The need for this technique surfaced from circumstances where fertilization with standard in vitro insemination fell short. The difference between the two being in the fact that standard in vitro insemination requires functionally normal spermatozoa, while most of cases of complete and unexpected fertilization failure were attributed to a putative male factor, or other unclear reasons. The seemingly more frequent inclination to immediately allocate couples to ICSI treatment without an indication for its utilization has now allowed the rise of a new problem—fertilization failure even with ICSI, the very outcome it was designed to prevent.

The qualm is that this comparison seems to favor standard in vitro insemination to mitigate skepticism surrounding ICSI, whether related to its overuse or its performance. ICSI is no better or worse than standard in vitro insemination, and in practices with a large population of couples plagued by male factor infertility, it makes sense to do mostly ICSI insemination. ICSI depends on characteristics of patient population, and the specific variables present in the laboratory.

We are not going to answer this question with this manuscript, or even in this issue. But one thing is certain—ICSI is here to stay.
